# Guidance for Autonomous Underwater Vehicles in Confined Semistructured Environments †

**DOI:** 10.3390/s20247237

**Published:** 2020-12-17

**Authors:** Zorana Milosevic, Ramon A. Suarez Fernandez, Sergio Dominguez, Claudio Rossi

**Affiliations:** Centre for Automation and Robotics UPM-CSIC, Universidad Politécnica de Madrid, 28006 Madrid, Spain; fernandez.suarez.ramon@gmail.com (R.A.S.F.); sergio.dominguez@upm.es (S.D.); claudio.rossi@upm.es (C.R.)

**Keywords:** underwater robots, guidance, 3D navigation, field robotics, software-in-the-loop, hardware-in-the-loop

## Abstract

In this work, we present the design, implementation, and testing of a guidance system for the UX-1 robot, a novel spherical underwater vehicle designed to explore and map flooded underground mines. For this purpose, it needs to navigate completely autonomously, as no communications are possible, in the 3D networks of tunnels of semistructured but unknown environments and gather various geoscientific data. First, the overall design concepts of the robot are presented. Then, the guidance system and its subsystems are explained. Finally, the system’s validation and integration with the rest of the UX-1 robot systems are presented. A series of experimental tests following the software-in-the-loop and the hardware-in-the-loop paradigms have been carried out, designed to simulate as closely as possible navigation in mine tunnel environments. The results obtained in these tests demonstrate the effectiveness of the guidance system and its proper integration with the rest of the systems of the robot, and validate the abilities of the UX-1 platform to perform complex missions in flooded mine environments.

## 1. Introduction

Europe is strongly dependent on raw material imports [1]. At the same time, there is a very high number of abandoned and potentially profitable mining sites in its territory. A recent survey on abandoned mines in Europe collected data regarding more than 8000 locations [2]. Moreover, the geological community suspects that there could be as many as 30,000 inoperative and potentially profitable mining sites.

Most of these sites were abandoned in the last century due to economic reasons rather than actual ore exhaustion. Nevertheless, with the latest advances in mining technology, it may be profitable to reopen some of these sites, a number of which may contain raw materials that are currently in critical demand [3] and whose exploitation would reduce Europe’s dependency on external sources.

The increasing interest in reopening some of these abandoned mine sites, however, faces a severe problem: lack of reliable, up-to-date information regarding the mines’ topology and structural soundness as well as mineralogical information. In fact, in many of these sites, mining ceased more than a century ago. The information regarding the structural layout of the tunnels is limited and imprecise or even totally lacking. Hence, to acquire the topological, structural, and geoscientific data, the core information for assessing the mine’s status, and the feasibility of reopening, initial surveys must be carried out.

It must be pointed out that under normal operation conditions, water must be continuously pumped out from the tunnels to maintain a safe working environment. Once the mine is permanently closed, the dewatering systems cease to operate, and the tunnels are slowly flooded.

Needless to say, the complex network of tunnels constitutes an extremely hazardous environment; prospecting by conventional methods such as human divers would place them in an extremely risky scenario, in addition to the fact that they can only reach a relatively limited depth range, much smaller than that of a standard mine [4]. The use of underwater vehicles (UVs) appears thus as a natural alternative to overcome the drawbacks of direct human exploration. Conventional Remotely Operated underwater Vehicle (ROV) technology is also ruled out due to the the need for a communication channel between the vehicle and its operator, which cannot be practically established using the existing methods due to the specific characteristics of the flooded mines. The use of a conventional tether would be unsuitable given the dimensions and complexity of the mines, since the weight that such length of cable would exert and, more importantly, the risk of entanglement in protruding elements of the walls of the mine it would cause could not be assumed. Additionally, the ad hoc self-deployment of a network based on a trail of nodes, e.g., breadcrumb networks [5] (see Section 2), could not be considered suitable either for flooded mine exploration for a number of reasons: first, the shape, size, and even topology of the tunnels and shafts of the mine is unknown a priori, which prevents the use of beacons with specifically tailored fixation means [6]; moreover, certain mines are in a precarious structural state, which discourages any contact with their walls due to the risk of collapse; also, the deployed nodes could represent, depending on their shape, obstacles for the movement of the submarine itself and an unacceptable alteration of the natural environment if left abandoned; and finally, they do not provide enough throughput for the bidirectional real-time communication required for the control of the operator. Given all these difficulties, the use of autonomous robots appears to be an ideal alternative and possibly the only suitable choice.

**Figure 1:**
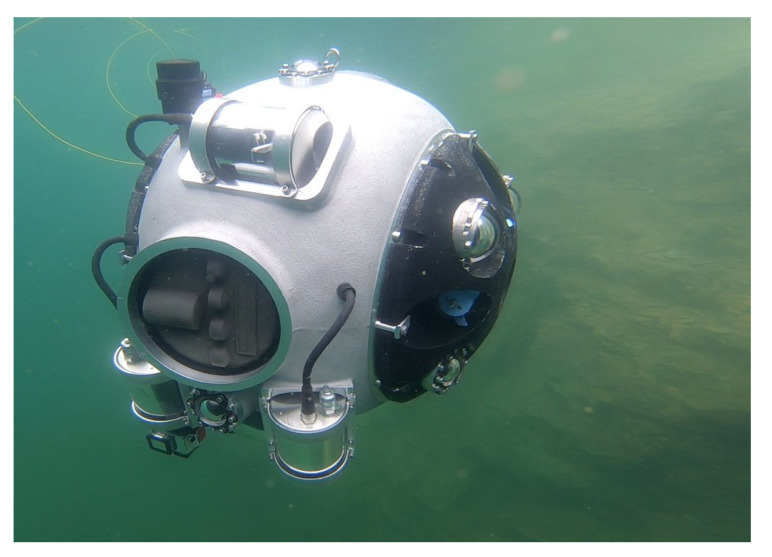
The UX-1 underwater vehicle during field trial in Kaatiala mine (Finland).

The UNEXMIN project (UNderwater EXplorer for flooded MINes) was born to answer these challenges. The main objective of the project is the design of a team of autonomous underwater vehicles (AUVs), named UX-1 class underwater explorers (depicted in Figure 1, and hereafter simply UX-1), specifically tailored to explore underground flooded mines and their testing in such environments; therefore, the UX-1 robots are equipped with navigation instrumentation that allows them to build a map of the mine as they go through the tunnels and collect geological information through the scientific instrumentation they are equipped with.

The main research goal of the work presented in this paper is the design and implementation of the autonomous guidance system of the novel UX-1 underwater robot and on its validation using test paradigms comprising different degrees of simulation, namely software-in-the-loop (SIL) and hardware-in-the-loop (HIL).

The guidance system is essential to achieve the intelligence and autonomy required to operate in flooded mines due to the inherent restrictions imposed by such environments, discussed above (and developed further in Section 2). Such autonomy, in addition, must rely exclusively on positioning and mapping systems entirely dependent on the sensors installed on-board, once accepted that the operation of the robot excludes the possibility of positioning using external sources of information; and it must flexibly account for the harsh environmental conditions, that is, unknown, semistructured, labyrinthine environments with the presence of clutter. Another specificity of the robot is its novel mechanical design (e.g., a variable pitch system designed especially for the UX-1 vehicle) and its distinctive on-board scientific instrumentation, aimed at gathering scientific samples: the coordination of such instrumentation with the movement of the submersible itself, ensuring that the strict positional requirements of the scientific sample capturing for each type of sensor are fulfilled, must be ensured by the guidance system of the platform. For these reasons, the design and implementation of the guidance system of the UX-1 constitute a unique research challenge.

This paper is the extension of a previously published work carried out by the same authors [7], extension in terms of (i)  the level of detail in which the different components of the guidance system are illustrated, (ii) the level of development of the guidance system, still under continuous development, and last, but foremost, (iii) the completeness of the presented sets of tests to prove the capabilities of the system.

The paper is organized as follows: Section 2 reviews related work in the field of UVs, with special emphasis on the subfield of autonomous navigation. Section 3 provides details of the design of the UX-1 robot, including its mechanical design and properties, its computing hardware and navigation and scientific instrumentation, and its overall software architecture, necessary to understand the specifics of the architecture and algorithms of the guidance system, the main focus of this work and subject of Section 4. Section 5 reports the tests performed to assess the effectiveness of the developed algorithms and to achieve its tuning, whereas the conclusions drawn from them and additional notes on the future steps on the development of the guidance system are presented in Section 6.

## 2. Related Works

Unmanned UVs have many potential applications in a variety of fields, such as ocean mining exploration [8], autonomous seafloor mapping [9,10], data gathering [11,12,13], maritime security [14], marine archaeology [15,16], and search and rescue [17].

Although underwater navigation is a relatively consolidated field of research, most of the research in this field addresses open water environments, or, alternatively, partially confined environments such as submarine canyons or water masses under layers of superficial ice [18,19]. These environments, although undoubtedly very challenging, show structural characteristics that do not represent stringent constraints regarding the shape, size, and maneuverability of the robot: this is reflected, for instance, on the forward-moving torpedo-shaped design [20] followed by the vast majority of corresponding works [21]. In open water or semiconstrained environments, in addition, it is possible to establish communication paths with surface stations and/or vehicles, providing the UV with different levels of assistance. In this line, and mostly through the use of a physical tether providing a high bidirectional throughput between the UV and the assisting platform and allowing the continuous or intermittent intervention of a human operator, ROVs or UVs with supervised autonomy, respectively, have been extensively used [18,22,23]; a fast communication channel with the computational resources of a surface station could also be used to derive part of the computational tasks of the UVs to the latter. Linked to this capacity to establish a communication channel, in this case an acoustic channel, with GPS-enabled surface platforms, the UVs can obtain accurate positioning using long or short baseline (LBL/SBL) techniques [19,24]. Following this approach, manual mapping of 500 m of a shallowly submerged cave system was successfully achieved by the Sparus AUV [25].

The volume of works in the literature addressing the exploration of confined flooded spaces such as caves and tunnels, relating mostly to marine archaeology and geological prospecting [26], narrows down dramatically, which serves as an indicator of the bigger technical challenges posed by such environments. These challenges reflect on three main aspects of the UVs tailored for them [27]: their constrained mechanical design relating both shape and size, and their necessary high maneuverability; their difficulties in acquiring detailed positional information; and their required autonomy. These three aspects, detailed further in the following paragraphs, shape drastically the works in this field.

The need for high maneuverability and positional control has been signaled in the literature with the departure from the forward-moving torpedo-shaped submersibles typical from open water exploration [20,21] to, often, ellipsoidal [28], spherical [23], or cubical [22] shapes, able to perform movements in six degrees of freedom (DOF) and with hovering capabilities: hence their generic name, hovering AUVs (HAUVs). Following this line of work, the DEPTHX UV was carried out the exploration and mapping of hot springs  [28]; the submersibles Sentry [29] and Mesobot [22] performed oceanographic surveys and observations of marine life; and U-CAT [26], IMOTUS-1 [23], and and SUNFISH [27] were used to autonomously explore and map archaeological sites, storage tanks, and underwater caves, respectively. These three latter works, which can be considered the closest existing works to our UX-1 robot not only in terms of their field of application but also regarding their guidance, are discussed further below. Bioinspired designs extending the capabilities of the HAUVs with additional motor skills have also been proposed: a good example is the ASRobot [30], a small amphibious spherical robot for exploration tasks in restricted environments with the addition of legs for crawling.

The acquisition of absolute positional information through GPS-enabled surface platforms or beacons, as done often in open water UV, is infeasible in an underwater maze of rocks due to the lack of a direct acoustic communication link; needless to say, radioelectric signals have no practical use underwater due to their severe attenuation. More complex networks performing underwater localization are based not only on the deployment of surface landmarks but also acoustic nodes mounted on the seafloor or reefs [19]: this need for planning and node mounting makes them unsuitable for the exploration of inaccessible confined flooded spaces based on them. An alternative to said planned node deployment is embodied by the so-called breadcrumb networks, where the nodes of the network are self-deployable by an agent as it traverses the environment under study [5]. They present, however, a number of difficulties for their deployment in the flooded mines subject of our work: first, the breadcrumbs would represent an significant additional payload for the carrying submersible, whose design restrictions are already highly limiting; second, the deployed nodes could represent, depending on their shape, obstacles for the movement of the submarine itself; and third but foremost, breadcrumbs for underwater applications would require fixation means, which could not be specifically tailored [6] for exploration missions given the lack of knowledge on the environment to explore and would additionally require contact with the walls of abandoned and flooded mines, often in a precarious structural state, which is strongly discouraged due to the risk of collapse.

Existing UVs for confined flooded spaces base therefore their positioning on inputs on their relative pose captured on-board, either with respect to their position in previous time steps like the inertial measurement units (IMUs), fiber-optic gyroscopes (FOGs), and Doppler velocity logs (DVLs) or with respect to their environment, like the sonar [23,26,27]. These inputs, partial and noisy, in the case of sonar because acoustic signals suffer from multipath in narrow and intricate pathways, are integrated using accumulative processing such as dead reckoning, Kalman filters, or Simultaneous Localization and Mapping (SLAM) techniques [21,26].

Not only a wireless link between the robot and external assistance is unfeasible in flooded mines composed of intricate labyrinthine passages, as detailed above: tethered communications are unfeasible too. First, achieving a neutrally buoyant tether in unknown, or only partially known, multilevel environments spanning hundreds of meters is impractical, and therefore the influence of the cable would represent a severe disturbance for the dynamics of the UV. Second and foremost, given the frequent clutter of the consider environments and their extension the risk of potential entanglement in protruding elements such as ladders, cranks and levers, or simply rocks is unaffordable. This lack of communication leaves the UV alone with its autonomous capabilities, both in terms of sensing and environmental mapping and of decision making [26]. The autonomy of a vehicle, at least in terms of displacement decisions, is greatly embodied in the concept of path planning [31], i.e., deciding which sequence of locations in the space to follow in order to reach a determined target location, which shows a tight interdependence with the representation of the known environment of the robot in the form of occupancy maps [26], ensembles of features [23], or different but analogous structures [27,32].

Underwater path planning has been often addressed in the literature as a 2D problem, analogous and interchangeable with terrestrial path planning [31]. In this direction, [33] discloses a framework for the autonomous exploration of confined, indoor environments sharing a number of key features with underwater tunnel exploration, since their topology can also grow in complexity, constructing a semantic road map that represents the topology and can be incrementally built. Certain related approaches, considering equivalence classes of maps and incorporating specifics of underwater navigation such as current forces have also been proposed [34]. However, the simplification into a flat 2D problem, although convenient, does not suffice for flooded mining environments, which require 3D exploration to capture tunnel and shaft structures. To this end, a variety of path search algorithms suitable for both 2D and 3D environments, both deterministic and probabilistic like rapid exploring random trees (RRT) [35], have been proposed in the literature [31].

All aspects considered, the HAUV systems U-CAT [26], IMOTUS-1 [23], and SUNFISH [27] are, in terms of their objectives, size, maneuverability and positional sensing, degree of autonomy, and path planning capabilities, the closest platforms to our UX-1 submersible. For instance, according to the authors of the SUNFISH platform, said robot is capable of “autonomous exploration in unstructured environments and was designed to be a highly-capable platform for operating in a wide variety of complex 3D spaces, ranging from man-made (e.g., piers or harbors) to natural (e.g., reefs or caves)”. The differences of our UX-1 robot and its guidance system derive, primarily, from our special interest in gathering, contactless, not only structural information on the site but also detailed geological data, which conditions both the payload of the platform and type of considered missions.

## 3. Details of the Robotic Platform UX-1

The comprehension of the presented guidance system, detailed in Section 4, requires certain understanding of the robotic platform UX-1 it is tailored to. For completeness, the following subsections provide an overview of the UX-1 submersible in terms of its mechanical capabilities, its computational capabilities, its navigation and scientific instrumentation, and general software architecture.

The special mechanical design of the UX-1 is the result of the extremely specific characteristics of the types of mission and environmental conditions that the UNEXMIN project targets at: high autonomy and maneuverability in flooded mines composed of intricate networks of narrow tunnels and shafts. Having such special restrictions into account, a spherical shape was eventually chosen for the UX-1 (see Figure 1), implemented as a machined aluminum pressure hull capable of enduring water pressure levels up to 50 bar, corresponding to a water depth of 500 m.

The size of the submersible, a diameter of 60 cm, represents the necessary balance between the expected minimum diameter of the narrower tunnels of the mines targeted by the project and the need for room, inside its hull, for the required navigation and scientific instrumentation. This latter point is essential, since all the equipment of the submarine (but for its thrusters) needs to be encased inside its hull for two reasons: first, the high water pressure at certain operational depths would otherwise harm elements outside the high-pressure hull; and second, mine tunnels and shafts usually contain elements protruding from their walls (e.g., metal bars, cables, ladders, and detached rocks) that could jeopardize the mission and thus endanger the robot itself if entangled with any element external to it.

The main characteristics of the UX-1 robot are outlined in Table 1. Further details on its mechanical design can be found in [36].

### 3.1. Mechanical Design

#### 3.1.1. Motion System

**Figure 2:**
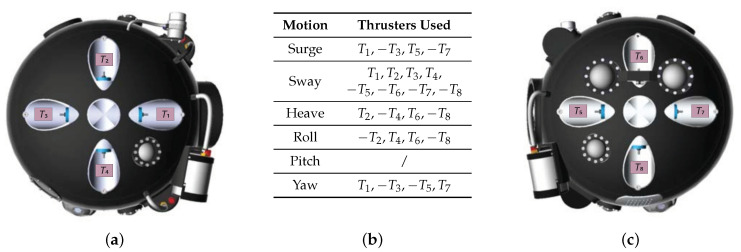
3D model of UX-1 robot and its thruster configuration. (**a**) Right view. (**b**) Thruster configuration. (**c**) Left view.

The spherical design of the UX-1, combined with its propulsion system composed of two sets of four thrusters placed, respectively, in a cross configuration at each side of the sphere (see Figure 2a,c), allow 5 DOF motion and translation movements in all directions regardless of the heading of the vehicle.

The configuration of actuators for the different motions is depicted in the table in Figure 2b. The mapping matrix **B** is used to define how the thruster configuration acts on the dynamics of the vehicle: given a reference force vector and inverting (i.e., Moore–Penrose inversion) the mapping matrix, we can compute the force required by each thruster. The mapping matrix is 6×8, wherein each column corresponds to a thruster and each row represents one of the 6 DOF, namely surge, sway, heave, roll, pitch, and yaw. Using the thruster configuration shown in Figure 2b, the matrix **B** is defined as

(1) $$B=\left[\begin{array}{cccccccc} 1 & 0 & -1 & 0 & 1 & 0 & -1 & 0\\ 1 & 1 & 1 & 1 & -1 & -1 & -1 & -1\\ 0 & 1 & 0 & -1 & 0 & 1 & 0 & -1\\ 0 & -l & 0 & l & 0 & l & 0 & -l\\ 0 & 0 & 0 & 0 & 0 & 0 & 0 & 0\\ l & 0 & -l & 0 & -l & 0 & l & 0 \end{array}\right]\;\;,$$

with

(2) $$l=\sin\left(\delta \right)\sqrt{\sigma_{1}^{2}+\sigma_{2}^{2}}\;\;,$$

where $\sigma_{1}$ is the distance from the axis of the thrusters to the geometrical center of the vehicle, $\sigma_{2}$ is the distance from each thruster to the middle lateral point, and $\delta =\arctan(\sigma_{2}/\sigma_{1})$ is the rotation angle of the moments generated on the robot. Note that the pitch movement is not achievable by the thrusters; therefore, its corresponding row in the mapping matrix is filled with zeros.

An additional degree of freedom is achieved by means of a variable pitch system that uses batteries as an inner pendulum, whose weight displacement generates, accordingly, the desired pitch angle in the submersible. The UX-1 robot is required to traverse up to 500 m vertically to explore deep mines. Continuous operation of thrusters would require a significant power consumption and would considerably decrease the desired operating time of 5 hours. To prevent this, a small sized variable ballast system was designed to allow active adjustment of the buoyancy during the long vertical movements. The complete theory and development of the ballast system can be found in [37].

### 3.2. Hardware and Sensors

The submersible has a distributed computer system for on-board data processing, sensor interfacing, and actuator control. Due to the size limitations of the hull’s interior, a Computer-on-Module (COM) Express Type 6 was chosen as the main computer responsible for the mission control. This computer performs multiple sensor integration and data fusion. Furthermore, it hosts the Guidance, Navigation, and Control (GNC) algorithms, and it is responsible for all sensor data registration and storage for further postprocessing. Additionally, COM Express Type 10 Dedicated CPUs are used in intensive specific sensor processing tasks.

**Figure 3:**
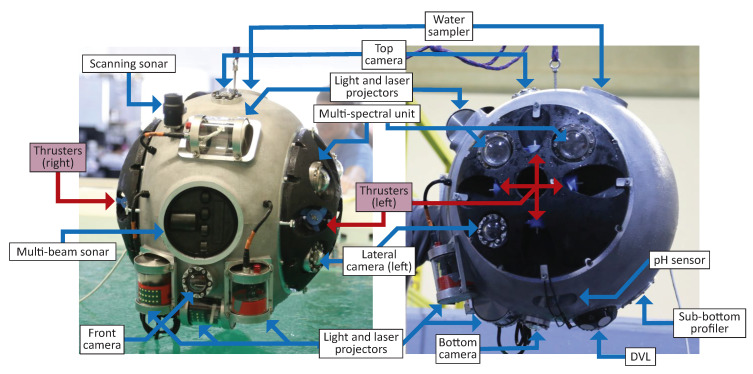
Scientific, navigation equipment, and thrusters of the UX-1 robot: left image, front view of the UX-1; right image, left side view.

Regarding the instrumentation incorporated by the robot, encased in the hull too and schematically showing in Figure 3, two separated categories can be established:

- Scientific instrumentation, exclusively for geological data collection and thus not used for the functioning of the robot itself but obtain samples from the mine. The sensors included by the robot are: thermometer and barometer, water sampler, conductivity and pH measuring units, sub-bottom profiler, magnetic field measuring unit, UV fluorescence imaging, gamma ray counter, and multispectral imaging unit.  
- Navigation equipment, necessary for the essential functions of the submersible and with a direct relevance to the guidance system further developed in this paper. The robot localization uses a fiber optic-based IMU (KVH 1750) for linear acceleration and angular velocity measurements. DVL (Nortek 1 MHz) is used to estimate the pressure, relative vehicle velocities, and distance to bottom measurements. These measurements are integrated over time using the dead-reckoning technique. A Multibeam Profiler Sonar (Kongsberg M3) generates imaging and bathymetric data, and it has a distance range of up to 500 m. Additionally, the robot has a mechanical scanning 360°; sonar (Tritech Micron) with up to 75 m range used mainly for obstacle detection. A custom-developed laser-based structured light systems provide detailed point cloud data and depth estimation: the Structured Light System (SLS) is comprised of five digital cameras with 110° lens opening, 9 fps, and 2054×1544 image resolution, a dedicated image processor CPU, and a light projector system. The light projector system has a visible light source, an UV light LED projector, and a rotating laser line projector.

Data acquired from both navigation and scientific instrumentation sensors are recorded in real-time and stored to disk. For this purpose, hardware interfaces have been implemented in C++ or Python in Ubuntu 16.04., and data communications are handled using the Robot Operating System (ROS) standard messages. ROS is chosen as a middleware (an abstraction layer between the hardware and the software) due to its modularity, good hardware support (drivers), and its messaging system that simplifies the communication between different software modules [38].

Further details on the hardware architecture of the UX-1 can be found in [39].

### 3.3. Software Architecture

**Figure 4:**
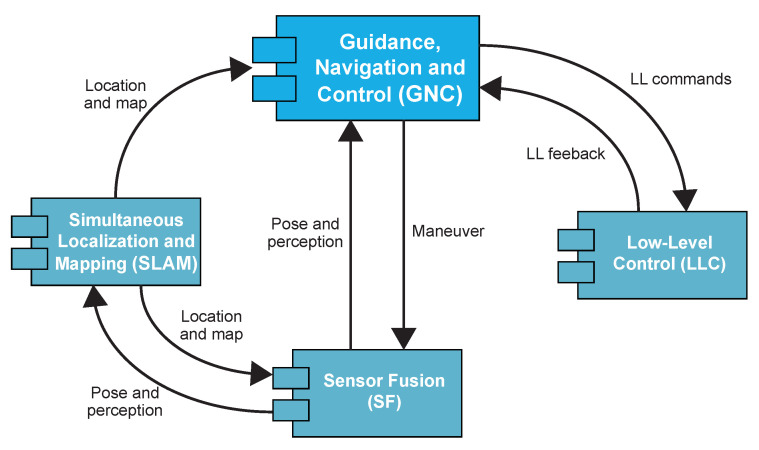
The UX-1 software architecture.

The software of the UX-1 robot has been designed following, as far as possible, a modular organization. This division into modules has, as its main benefit, a high degree of independence between modules as far as their defined interfaces are respected: this independence allows their fairly independent development, something important in an international project like UNEXMIN where the modules are developed by different teams based in different countries and enables an almost straightforward modification and testing of each module separately with minimal effect on the rest of the modules. As a result, the architecture of the UX-1 consists of four main modules: Sensor Fusion (SF), Simultaneous Localization and Mapping (SLAM), Low-Level Control (LLC), and Guidance, Navigation and Control (GNC) (see Figure 4).

The SF and SLAM modules, developed by researchers at INESC-TEC, provide the GNC system with localization information (pose, global localization, and map data). Based on this information, the GNC module is in charge of generating waypoints and trajectories to be followed by the robot and controlling that the actual motion of the robot attains this planning. In the SF, a 3D mapping node integrates the point cloud information obtained by the Tritech Scanning Sonar, the M3 Multibeam Sonar, and the SLS. Additionally, the SF module implements an Adaptive Extended Kalman Filter to estimate the position, velocity, and attitude of the UX-1 robot by using data provided by DVL, IMU, and pressure sensor. Finally, the SLAM module receives the pose information and the mapping data and provides a corrected 3D map and global localization estimates. The LLC module, implementing different approaches of control theory to directly affect the actuators using low-level commands, shows a simpler architectural structure composed of two monolithic submodules. For further details regarding the SF and SLAM modules, we refer the reader to [40], and for LLC module, we refer the reader to [36].

The GNC module is composed of the three submodules suggested by their name. According to [41], the GNC’s submodule can be defined as:

- Guidance is the system that continuously computes the reference (desired) position, velocity, and acceleration of a vehicle to be used by the motion control system. Sophisticated features such as obstacle avoidance and mission planning can be incorporated into the design of guidance systems.
- Navigation is the system that uses available sensors to determine the submersible’s state, its position/attitude, velocity, and acceleration.
- Control, or more specifically, motion control, is the system determining the necessary control forces and moments to be provided by the submersible to satisfy a particular control objective.

**Figure 5:**
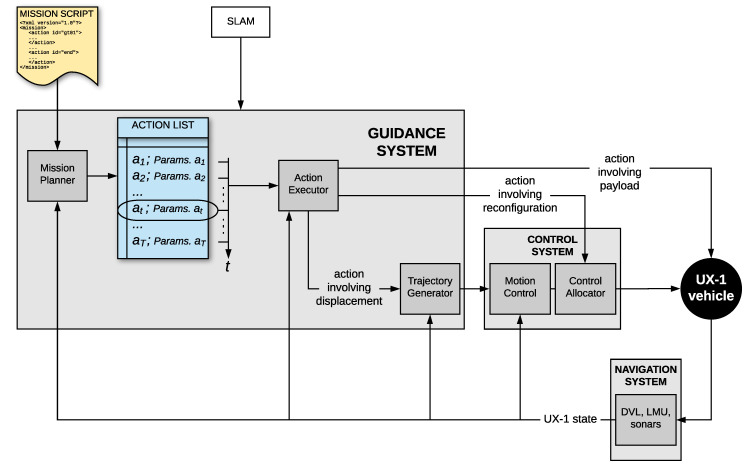
Block diagram of the operation of the Guidance, Navigation, and Control (GNC) module.

These three submodules of the UX-1 robot interact with each other through data and signal transmission, as illustrated in Figure 5.

The autonomous guidance system, which represents this paper’s main focus, is described in detail in the next sections. For details regarding the Navigation system, we refer the reader to [40], and regarding the Control system, we refer the reader to [42,43,44].

## 4. Guidance System

The following subsections describe with details the implemented guidance system through the dissection of its component subsystems: Mission Planner, Action Executor, and Trajectory Generator. The current architecture, described in the following subsections in reference to Figure 5, is the evolution of the guidance system as presented in our previous works (compare with [7]) as a result of successive redesigns motivated by corresponding tests.

### 4.1. Mission Planner

The Mission Planner module is in charge of configuring the overall mission strategy, defined as a list of high-level, semantic tasks which include, whenever relevant, the parameters needed to specify them (e.g., in scientific instrumentation measurement tasks, the map locations they apply to). Those tasks will be referred to as *actions*.

The Mission Planner takes as an input a set of actions desired by the operator, defined in an order related to their intended temporal precedence in the form of an XML-based mission script. Such script is read, interpreted, and possibly complemented with additional auxiliary, operative actions to create an ordered action list (see Figure 5); the meaning of such operative tasks will be detailed below, along with the rest of the scientific and exploratory actions explicitly definable in the mission script (Section 4.2).

The Mission Planner and the resulting action list are of a dynamic nature: actions from the existing list are sequentially read and dealt with one by one; upon the termination of the current task, and depending on its result or on the resulting state of the submersible, the Mission Planner can perform a modification of the remaining actions of the list. The most intuitive dynamic modification of the action list would correspond to the detection of a low battery level of the submarine, which would result in the cancellation of the remaining actions and their substitution for an immediate return to a safe location or starting point (see Section 4.2.3).

### 4.2. Action Executor

The main goal of the Action Executor module is accepting actions from the action list generated by the Mission Planner and executing them.

Regarding their purpose and scope of use, actions can be classified into one of these four types: *pure displacement*, *scientific sampling*, *exploration*, and *event handling*. Both pure displacement and scientific sampling actions are relevant for those areas of the mine whose map is known and available. In contrast, exploration actions aim to obtain the geometric information of unexplored sections of the mine and incorporate it into the map of the environment. Event handling actions, however, correspond to situations where disruptions in the correct performance of the submersible are detected or foreseen and where, as a consequence, exceptional measures to preserve its integrity or safety need to be taken.

Pure displacement actions, simply responding to the submarine’s desired destination position within a known section of the mine indicated by the mission script, require no further description. Contrarily, scientific sampling, exploration, and event handling actions are illustrated further in Section 4.2.1, Section 4.2.2 and Section 4.2.3, respectively.

#### 4.2.1. Scientific Sampling Actions

The UX-1 robot utilizes various sensors for gathering geoscientific data (see Section 3.2). The efficient data logging capabilities of ROS make it useful to record and store sensory information. By having the sensors drivers implemented on ROS, we can publish data in a defined format for each sensor and append the positional and time information.

Each of the available scientific sensors has its own set of reconfigurable parameters. Some of those parameters do not affect the mission’s execution, e.g., the sampling frequency of a pH sensor. However, the operation of some scientific sensors entails certain positional requirements for the robot: a good example of this is the collection of data using the multispectral unit, which requires keeping a certain distance to the mine wall while moving at a constant velocity during the operation. Table 2 summarizes the scientific sensors and their corresponding parameters.

Before the mission, scientific sampling actions, defined by the user, are inserted in the mission script together with their parameters, including, when applicable, their desired location, time, or both: the Action Executor handles the different aspects involved in each specific sampling action by engaging the required corresponding modules, as depicted in Figure 5.

#### 4.2.2. Exploration Actions

Due to the nature of most mines’ geometry, normally organized in horizontal layers connected by the vertical shaft, we can simplify three-dimensional exploration by decoupling horizontal plane and vertical exploration:

##### Horizontal Exploration

Horizontal exploration is used for traversing tunnels. The approach we used is based on the detection of frontiers [45], regions on the border between explored and unexplored space. During exploration, a robot navigates to successive frontiers to extend the known area until the complete, reachable environment is explored.

Our exploration has been based on the available ROS package frontier_exploration [46], which returns a list of frontiers which we further sort according to a *cost*, defined through the function

(3) $$cost\left(f\right)\;\;=\;\;w_{d}\;d\left(f\right)\;\;+\;\;w_{\Delta \theta }\;\Delta \theta \left(f\right)\;-\;w_{A}\;A\left(f\right)\;,$$

where *d* represents the distance to the frontier *f*, Δθ represents the required change in orientation of the robot to face *f*, and *A* is the extent of *f*, and where $w_{d}$, $w_{\Delta \theta }$, and $w_{A}$ are factors weighing the relative importance assigned to the different aspects. Consequently, the frontier in the current list of open frontiers that involves the lowest cost is chosen.

Furthermore, we merged the frontier exploration with the exploration strategy named wall-following logic [45]. Wall following logic, also known as the right-hand rule (or, analogously, the left-hand rule), is the best-known rule for traversing mazes [47]. Assuming that the walls are connected, then by keeping one hand in contact with one wall of the maze, the solver is guaranteed not to get lost and return to the entrance. We use the right-hand rule for horizontal exploration, a policy that is naturally enforced by appropriately setting the parameter $w_{\Delta \theta }$ of the cost function in Equation (3), conveniently signed, to favor the selection of frontiers involving Δθ towards the right.

##### Vertical Exploration

Vertical exploration is used for descending shafts. For initializing the exploration, the UX-1 robot must be placed in the center of the shaft cross-section. Then we use the depth readings of the DVL sensor for descending the shaft while keeping the position in its center.

Exploration actions’ entries in the mission script are thus comprised of the following parameters: desired duration of the exploration; for horizontal exploration: spatial coverage and/or a desired number of junctions to be entered; for vertical exploration: maximum depth to be reached if no shaft bottom is reached before.

#### 4.2.3. Event Handling Actions

In order to monitor changes in the world state, we adopt the concept of an *event*. This concept is broadly used in many automation programming languages, such as PLEXIL [48], a NASA’s Plan Execution Interchange Language used in autonomous spacecraft operations.

There are two changes in the world state that we want to monitor and handle:

##### External Events

External events represent a change in the external world’s state, such as a new obstacle appearing. They affect, above all, path planning.

**Figure 6:**
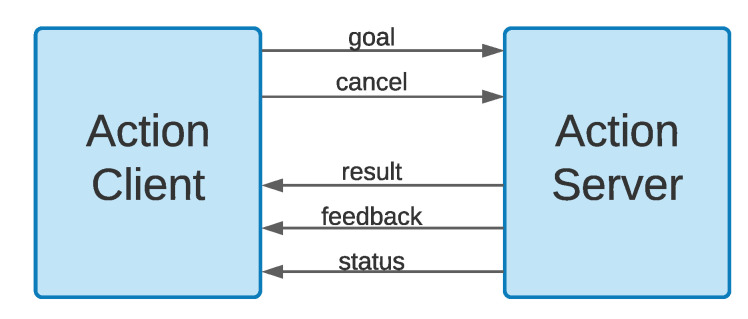
Robot Operating System (ROS) action interface.

We used the ROS action interface to track the status of the path execution. The actionlib [49] ROS package provides tools for creating servers and clients that execute long-running goals that can be pre-empted. In our example of path execution, a goal represents reaching a target location. A client and a server communicate using a predefined protocol that relies on ROS topics to transport messages (see Figure 6). A client sends a goal, e.g., moving to a certain target location, to a server through the *goal* message. Once a goal is received, the server creates a state machine to track the goal execution status and continuously notifies the client of the current state through the *status* message. When a new obstacle is detected and the current path is not feasible any longer, the goal’s state changes from valid to invalid, and the client sends a *cancel* request to the server. Subsequently, the client sends a request for a new path, and the server attempts to plan it. If replanning cannot generate a feasible path to the goal location, the robot is sent back to the base.

##### Internal Events

Internal events represent a change in the internal robot’s state. The internal events addressed here include low battery level; mechanical component failure (i.e., broken thruster); failure of sensors used for localization; and scientific instrumentation failure.

Internal events can be categorized into critical and noncritical events. The division is based on whether the detected event is jeopardizing the whole mission or not.

UX-1 robot is equipped with several scientific instruments (see Section 4.2.1) that are not necessarily used in all missions. Their usage is dependent on the type of mine being explored and on the user requirements. Hence possible faults of these sensors are handled taking into consideration the aforementioned needs. If the mission is focused on the exploration and mapping, the scientific instrumentation’s fault is not considered a critical failure; therefore, the mission is continued despite the failure. Nonetheless, if the mission’s focus is gathering the scientific data, the failure of the respective sensor is considered critical, and the mission is aborted.

The UX-1 is an overactuated system, where the number of actuators used to perform a control action is redundant [42]. Having that in mind, if one thruster fails, we can continue the mission by reconfiguring the force distribution for the rest of the thrusters. Therefore, a failure of only one thruster is considered a noncritical event, whereas a failure of more than one is considered a critical event.

A naive way of dealing with one thruster’s failure is disabling the symmetric thruster, i.e., the thruster in the same position in the hull but on the opposite side of the robot. Accordingly, the initial threshold for the low-battery is lowered to half its value: this modification is done to compensate for the new limitation in the robot’s speed caused by the loss of two thrusters, which would produce less distance coverage, hence possibly insufficient battery power to return to the deployment location. Therefore, we should abort the mission and signal the return to the base with a higher battery percentage compared to the situation when having all eight thrusters.

The disabling of the thruster is performed by changing the force allocation of the UX-1 robot. The force allocation control approach is based on the mapping matrix (see Section 3.1.1) that describes the relationship between the control inputs and the forces actually produced by the actuators. Knowing that the columns of the matrix correspond to each thruster, by changing their values we can manipulate the forces that each thruster must exert to match the given desired movement. Disabling a thruster is reflected by simply putting zero values in its corresponding column.

The approach of disabling the symmetric thruster would imply an almost immediate system adaptation and quick resumption of the mission. However, more sophisticated comprising self-diagnosis and self-adaptation capabilities would be desirable.

For this purpose, a metacontroller, a higher level AI-based supervision system capable of reasoning about the robot’s status, has been developed: instead of merely disabling symmetric thruster, the metacontroller would compute at runtime the optimal configuration of the rest of the thrusters. It was demonstrated that this approach provides an effective way to achieve system reconfiguration for achieving predefined goals. The specifics and testing of such controller are however not addressed in this paper: we simply refer the reader to [50] for further details.

Failure of navigation equipment necessary for essential robotic functions is considered a critical failure, and its handling is out of the scope of this paper.

### 4.3. Trajectory Generator

The Trajectory Generator module receives the desired goal location, generated by the Action Executor. Given the situation awareness and the given planning references, the Trajectory Generator module generates optimal collision-free motion references. The Trajectory Generator must include the knowledge of the robot’s dynamics and its actuation system constraints.

Often, time is not included as a planning restriction. Planning that takes into account only positional references is known as path planning. This simplification is only valid for specific tasks, i.e., mapping. However, it cannot be applied to applications requiring aggressive maneuvers for which velocity restrictions must be considered.

Trajectory Generator must handle uncertainties, minimize the possibility of collisions, be generic with respect to different maps, and find the optimum path in minimum time.

We adopted *MoveIt!* Motion Planning Framework [51,52] as a backbone for the development of our software. MoveIt! is a state-of-the-art software framework that provides an extensive functionality of features including motion planning, manipulation, kinematics, control, 3D perception, and collision checking. It has integrated widely used third-party libraries, including the Open Motion Planning Library (OMPL) [53] and A Flexible Collision Library (FCL) [54]. To represent the environment, we use an OctoMap library [55] that builds a 3D map based on an octree, an object shape supported by the FLC.

The path planning algorithm we employ is RRT-Connect [56], a bidirectional version of the Rapidly-Exploring Random Tree (RRT) algorithm [57] that builds two trees that grow towards each other, departing from the two way-points (source, destination). This algorithm was designed specifically for path planning problems with no limitation of the robot’s mobility due to the constrained dynamics of its motion (differential constraints).

## 5. Experimental Evaluation

### 5.1. Experimental Setup

The development and field testing of autonomous robots are complex tasks for a number of reasons. The logistics are often quite complicated, and the risk of damaging the equipment is very high. Such difficulties are greatly magnified in the development and testing of the underwater platform UX-1, the object of our work. The unique field working conditions of the UX-1, i.e., flooded subterranean mines, involve a high risk of permanent loss of the platform in case of failure or error and a high life risk in rescue attempts entailing direct human actions.

An additional difficulty during the development and testing of certain modules is the dependency on the existence and performance of the modules, hardware, and software, composing the loop they are part of. The guidance system is part of a closed-loop composed of the GNC module, the SF and SLAM modules, the LLC (see Figure 4), and, as important as them, the physical environment of the robot and the robot itself.

On the contrary, simulators are useful tools for system preliminary validation and algorithm benchmarking, where troubleshooting is simplified through the use of a controlled environment. Modern simulators have achieved astounding completeness and are able to simulate, with a high degree of realism, not only object dynamics but also all the operational components of a robotic system such as their sensor readings and communication protocols. Gazebo [58], for instance, is an open-source robotic simulator that has become the de-facto standard in the robotics scientific community, which includes multiple high-performance physics engines providing collisions and realistic movements, along with sensor data generation for a wide range of modalities.

Having in mind the above difficulties for autonomous robot testing and the capabilities of modern simulation software, we have developed an incremental testing framework for our guidance system based on the completion of the loop it is part of, discussed in the previous paragraph, using partly real and partly simulated modules and environmental data. In other words: our testing framework for the guidance augments, using different degrees of simulation, the real working modules, and the real data captured by the submarine on its environment, so all the interactions required for the guidance to operate are available.

**Figure 7:**
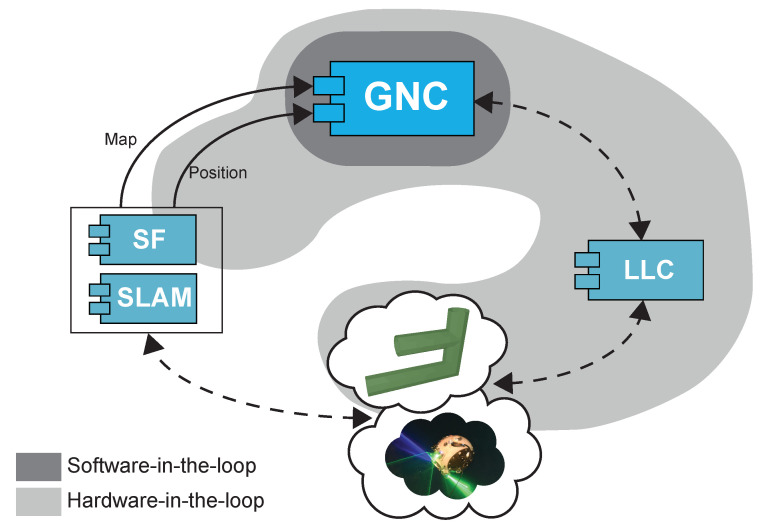
Visual depiction of the SIL and HIL testing paradigms.

Following this designed testing framework, two different degrees of simulation have been explored in detail: the first level is referred to as *software-in-the-loop*, or simply *SIL*, since all the components and data beyond the guidance and control systems are either purely software or simulated, allowing complete control of all the surrounding variables of interest; the second level is referred to, as a contrast, as *hardware-in-the-loop*, or simply *HIL*, because it incorporates the real hardware and software modules related to the control of the robot, the movement of the real robot in water, and the sensory readings of the robot regarding its positioning. Both testing paradigms are depicted and compared in Figure 7.

Both SIL and HIL tests, explained further in Section 5.1.2 and Section 5.1.3, respectively, simulate the presence of the robot in the mine, so the difficulties derived from the logistics and risks of on-site tests are avoided. However, in order to make the tests as representative as possible, realistic mine sites have been created and used in both testing paradigms, as detailed in Section 5.1.1.

#### 5.1.1. Virtual Environments

**Figure 8:**
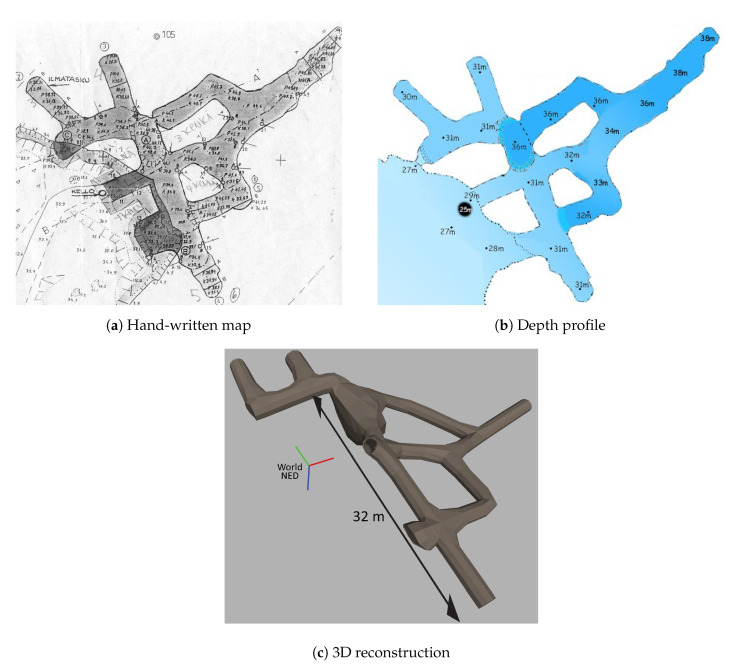
Kaatiala mine, Finland.

**Figure 9:**
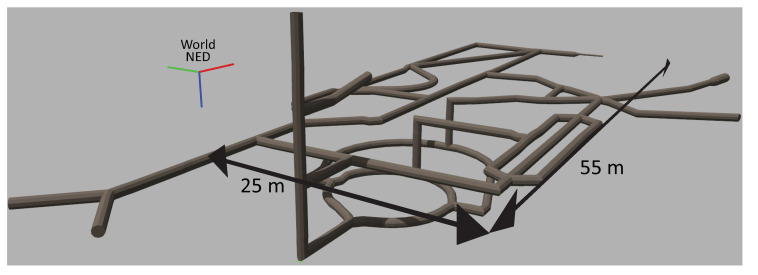
Complex virtual environment.

Several virtual environments, with different degrees of realism with respect to real underwater mines, have been developed to test different functionalities of the guidance system. The complexity of the simulated environments range from simple straight pipe-like cylinders in different orientations, adding progressively more obstacles inside them (see e.g., Section 5.2) and turns and joints of several angles and types, to the following two complete mine models:

- A model reproducing an existing mine, namely the Kaatiala mine in Finland, where the first field tests of the UNEXMIN project were performed. Kaatiala is an open-pit mine with an underground section currently being used for cave diving, for which an approximate layout and depth profile measured by the divers is available, as depicted in Figure 8b. The model of the mine, shown in Figure 8c, was artificially created using such limited low-detailed available information. This figure includes an indication of its *North*-*East*-*Down* (NED) frame of reference, where its axes are depicted, respectively, using red, green, and blue (RGB) colors: this convention will be kept in the rest of the paper.
- A purely synthetic complete mine, not corresponding to specific real data of any mine but only following general shape and size characteristics of real mine sections. It reflects a significantly more intricate layout than the Kaatiala model, with multiple bifurcations and junctions of different typesand useful for testing the capabilities of the Trajectory Generator and our exploration algorithm. A depiction of such a synthetic environment is shown in Figure 9.

The two environments representing full-size mines described above have been only used, however, in the SIL tests, since the size of the virtual environments used for the HIL tests are limited by the size of the physical tank in which the robot is physically operating.

#### 5.1.2. Software-in-the-Loop Setup

SIL experiments involve uniquely software, since the loop in which the guidance system is comprised is completed using purely simulations. They are thus performed without the participation of the real robotic platform itself, which is substituted for a realistic simulation model, including the environment and their mutual interactions.

Performing the SIL tests requires the simulation of, at least, the physical substrate of the modules SLAM, SF, and LLC (see Figure 4 and Figure 7) in operation with its environment, which includes: sensor readings (control commands and their effect on the position of the robot) and the environment itself. The modeling of the robot dynamics in the environment and the capture of readings from it have been simulated using Gazebo. The operation of the SLAM, SF, and LLC modules have been reproduced using different levels of idealization, ranging from the idealization of said modules facilitated by the availability of the ground truth provided by the simulation of the robot in the virtual environment, at their simplest level, to the use of the real software modules developed by their corresponding development teams and operating on realistic sensor capture simulations provided by Gazebo, at the most complex level of SIL experimentation.

Early testing SIL experiments present a number of additional advantages derived from the lack of limitations that both the robotic hardware and physical reality would impose on testing, to wit:

1. Detachment from the real passage of time: since simulations are not required to map simulation and real-time one-to-one, tests symbolizing a long test duration can be run in almost negligible time.
2. Full control of testing conditions: exact initial conditions for each test can be set and reproduced for subsequent tests, and different aspects inside/outside the scope of study of a certain experiment can be included/left out selectively.
3. Isolation of the effect of individual aspects in the performance of the guidance system: since the different modules of the tested loop run purely in the form of software, they can be selectively transformed into their ideal, flawless versions, allowing the consideration of realistic performance considerations for a limited number of them.

For instance, the above advantages, combined, have made it possible to develop sets of tests aimed at evaluating stochastic algorithms and experimental conditions through the statistical analysis of a significant amount of test runs (*1*) with identical starting conditions (*2*) and the optimization of the performance of the designed guidance system for certain operational tasks (*3*).

#### 5.1.3. Hardware-in-the-Loop Setup

HIL tests use both the real hardware and software of the UX-1 submarine, including controlling its movement in the water. The submarine is deployed in a water pool, where it dives according to the instructions provided by the LLC of the robot applied to the real robot’s dynamics. Real readings related to its positioning, such as depth, orientation, and velocity, are used for navigation and control purposes.

HIL experiments require, thus, the simulation of the solid walls of the mine in coordination with the real control of the robot in the pool, which involves synchronization between simulation and reality in two opposite directions of information: *(i)* the real displacement of the robot has to be reflected in the simulated environment so the virtual avatar of the submarine moves, accordingly, with respect to the walls of the simulated mine; and *(ii)* the sensor readings that the virtual avatar of the submarine would capture, from its position, from the simulated walls of the mine has to be transferred as the input for the corresponding sensors of the robot. According to these required communications, sensor readings related to robot positioning through the iterative creation of the map and used by the SLAM module, namely the SLS, the Multibeam Profiler Sonar, and the Scanning Sonar (see Section 3.2), were simulated in Gazebo and the real sensors were bypassed. The IMU and DVL, however, were not simulated, and their readings were real. The controller used for HIL experiments is the Feedback Linearization (FL) controller developed for the UX-1 platform and presented and validated in detail in [42].

**Figure 10:**
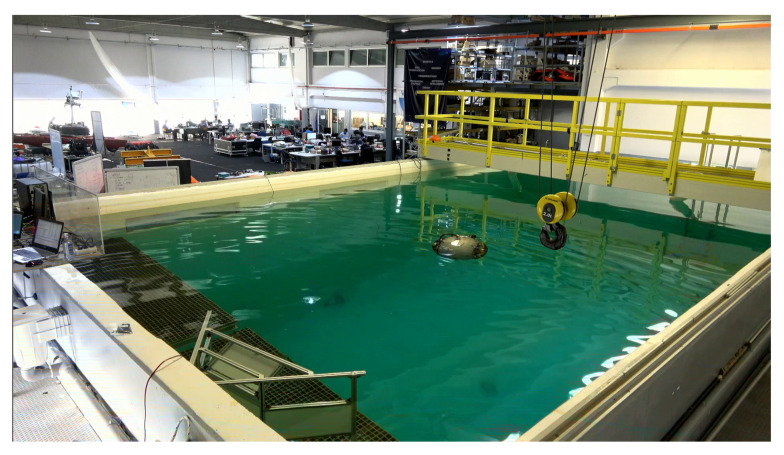
UX-1 in the water tank used for HIL tests.

It can be clearly seen that the extension of the HIL experiments is limited by both the amplitude of the virtual mine and the physical dimensions of the available pool. Our tests were performed in a 10×6×5 m (length, width, depth) water tank located in the Robotics and Autonomous Systems Laboratory at INESC TEC, shown in Figure 10. Since the tank size was far smaller than the dimensions of the two complete mine models discussed in Section 5.1.1, HIL tests could not be performed using a significant extension of their maps. Instead, different maneuvers covering partial aspects of a submarine campaign, such as shaft descent, tunnel entrance and traversing, and bend turning, were tested independently using limited corresponding models or sections of the complete mines.

**Figure 11:**
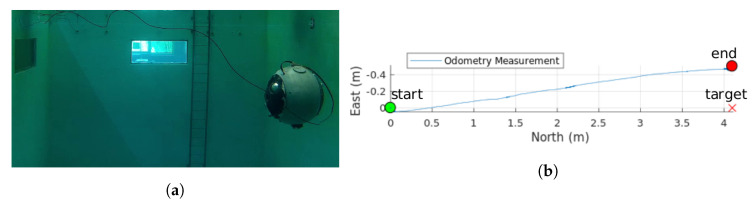
Drift in *East* (*y*) direction caused by the tether (**a**) UX-1 with the tether; (**b**) Odometry Measurement.

Although the UX-1 robot was designed and intended for fully autonomous navigation of flooded mines, during this preliminary test phase, the UX-1 robot was equipped with a neutrally buoyant cable for two reasons: first, real-time data communication regarding the simulation was required, as well as for visualization and parameter tuning; and second, the possibility of manual control in case of software or hardware failure needed to be granted. The addition of this cable, unfortunately, had a non-negligible effect on the robot dynamics, mainly in terms of lateral displacements (see Figure 11).

### 5.2. Performed Experiments

A series of experiments aimed at validating the guidance system’s performance in different tasks of interest for the submarine operation were carried out. Such experiments, performed, respectively, following only SIL, only HIL, or both paradigms depending on the specific task’s requirements, are detailed in the following subsections.

#### 5.2.1. Path Planning Experiments: Obstacle-Free

The objective of the performed path planning tests consists in following, in an open-water map (free from walls and obstacles), a path preset using a number of intermediate waypoints entered sequentially: the expected result of this experiment is, thus, a piece-wise linear trajectory between waypoints.

The assessment of the accomplishment of the tasks was based uniquely on spatial considerations, that is, the fact that the sequential waypoints were successfully reached within a certain spatial tolerance; no temporal restrictions regarding the time required for completion were evaluated. The experiment for a given path to test, defined as a list of waypoints, consisted thus in: transmission of the next waypoint to reach to the Trajectory Generator module; calculation, by the Trajectory Generator module, of the path to follow from the current position of the submarine to said waypoint; and, once the goal has been reached within the preset tolerance, repetition of the same steps using the next waypoint in the list.

Waypoints comprised both the desired location in space and the orientation of the submersible, and were entered following the format [x,y,z,ϕ,ψ], where the location variables *x*, *y*, and *z* follow the usual *North* (*x*)-*East* (*y*)-*Down* (*z*) convention for marine navigation, and ϕ and ψ represent, respectively, the pitch and yaw of the robot. (Note that no roll has been included since it has been considered of little practical interest.) The instant pose of the robot during the tests was recorded using the same coordinate system detailed for the waypoints. The tolerance for waypoint reach was preset independently for each dimension of the coordinate system: the tolerance for the translation-related dimensions *x*, *y*, and *z* was set to 10 cm and for the orientation-related dimensions ϕ and ψ to 5 degrees.

**Figure 12:**
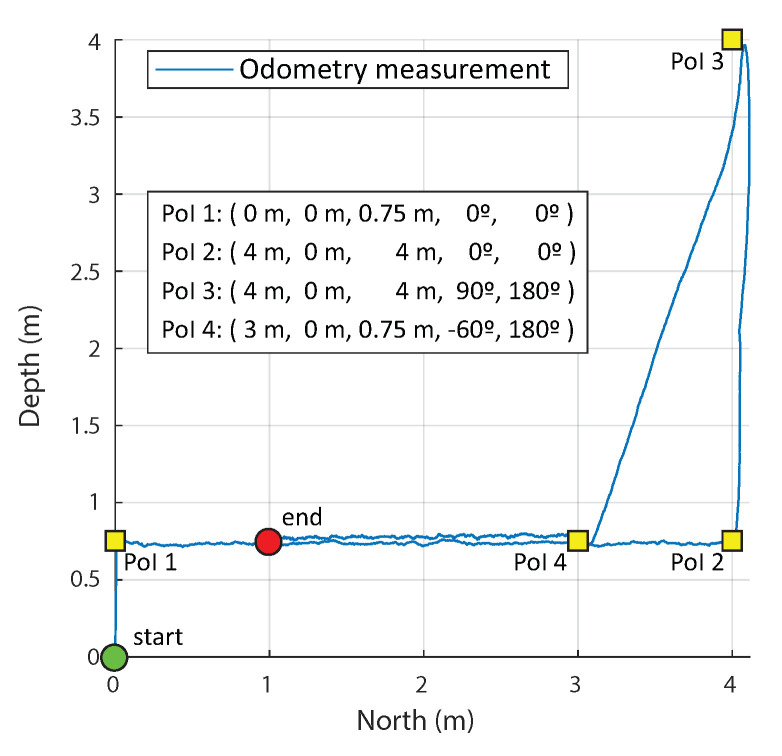
Waypoint control results.

The performed path planning experiments comprise both SIL and HIL tests with open-water maps (equivalently, no maps). Figure 12 depicts the results of one of the HIL path planning experiments, where the waypoints of the desired path are linked to four points of interest (PoI), which could correspond to a situation where the submarine traverses a horizontal tunnel connected to a narrow, vertical shaft extending downwards (although, as explained, no map including walls to be considered by the guidance system are included). The defined PoIs would correspond, in this symbolic setting, to: PoI 1, entrance of the tunnel; PoI 2, entrance of the shaft, end wall in the forward direction; PoI 3, bottom of the shaft, descending vertically by its end wall from PoI 2; and PoI 4, connection of the horizontal tunnel and the narrow shaft. Each point was sent to the path planner, which planned the safe path from the starting point to the goal: these points and the real positions described over time by the robot in the water pool appear marked in Figure 12. The inclusion of the separated PoI 2 and PoI 4, instead of one single point reached twice, was specifically considered to illustrate that the path planner correctly handles paths not aligned with the axis of the considered coordinate system but also oblique lines whenever suitable. Furthermore, the pitch angle was set to be different in the different waypoints: Nose Down configuration (90°) was part of PoI 3, and thus was used while descending, and the Nose Up configuration −60° was part of PoI 4 and thus was used while ascending the shaft diagonally. Points PoI 1, PoI 2, and the endpoint were set according to the Nose Front configuration (0°), and thus such configuration was used while moving in a straight line and exploring the horizontal mine passages.

The parameters of the RRT-Connect path planner were set knowing that the experiment is performed in an obstacle-free environment. The discretization of the robot motion was set to 20 cm, the planning time was limited to 5 s, and the maximum number of planning attempts was set to 5. The waypoint control experiment had a duration of approximately 545 s. The root-mean-square deviation (RMSD) in *x* and *z* direction is 0.056 m. The total length of the reference path is 13.4 m, and the total calculated traveled distance is 15.1 m. This difference could be due to the noise in the odometry measurement. In the water tank where this experiment was performed, no external positioning methods were available; therefore, the position of the robot was calculated fully internally by the robot’s SF module. This experiment demonstrated a successful execution of the guidance module and its proper integration with the SF and LLC modules. Table 3 shows the mean and max force reference commands obtained by the FL controller, the length of the reference and the accomplished path, RMSD of vehicle’s position with respect to the planned path, and the duration of the experiment. The planning time is not considered critical for our case due to the low velocity of the submersible, and therefore it has not been evaluated.

During these initial experiments, the sway motion was not controlled. Therefore, the drift caused by the tether could not be corrected. Figure 11b depicts the drift that occurred during the execution of this experiment.

#### 5.2.2. Path Planning Experiments: Environmental Restrictions

Closely linked to the obstacle-free path planning experiments described in Section 5.2.1 and as an incremental level of difficulty over them, the ability of the guidance system to plan paths adapting suitably to the environmental map and potential obstacles within the tunnels and shafts was tested.

Two types of obstacles were included in the considered environment models: (1) virtual mine walls to test maneuvers for mine traversing and (2) virtual obstacles inside the mine to test more challenging scenarios. The goals were predefined in an analogous manner to the waypoints detailed in Section 5.2.1 but, in the current setting, the path planner is assumed to infer more complex maneuvers inside the provided tunnels and shafts to achieve the goals without collisions. This type of experiment was performed both in SIL and HIL environments. However, for the HIL tests, the size of the pool greatly limited the virtual environments which could be considered; as a result of this limitation, we tested different maneuvers, such as descending the shaft and entering tunnels, traversing the tunnel and taking a turn, to name a few, in reduced combinations fitting the available pool size.

**Figure 13:**
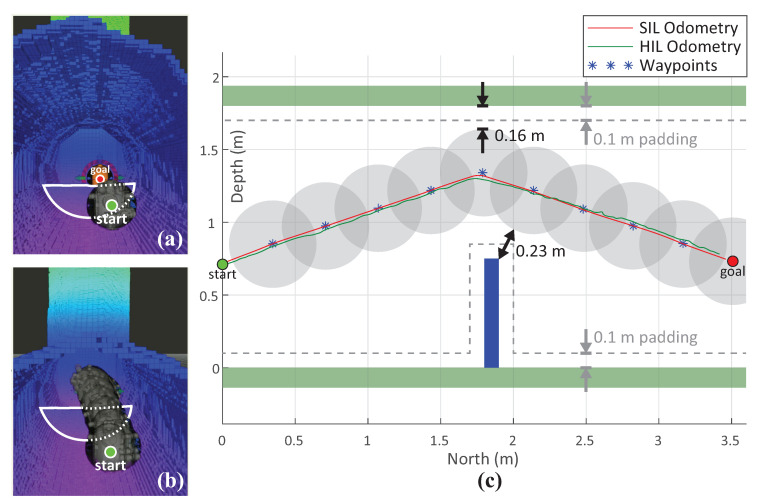
Path planning experiment with environmental restrictions depicted in different views. (**a**) OctoMap of the environment with start and goal position of the robot. (**b**) 3D planned path. (**c**) Side view of the mine tunnel with an obstacle between start and goal position of the robot. Tunnel walls are depicted in green, the obstacle in blue, and gray circles depicts the planned path.

Here we present the results of one type of maneuver tested independently in SIL and HIL environments. The experiment consisted of a narrow horizontal tunnel with a sharp narrowing caused by an obstructing wall in its lower half. The experiments in SIL and HIL were performed with the identical parameters in order to better evaluate their performance and compare them. Figure 13 depicts the results of the performed maneuver. Figure 13a represents the initial position of the UX-1 robot and the desired destination. Note the obstacle between these two positions, which was chosen intentionally to test the obstacle avoidance algorithm.

The OctoMap was prebuilt and it was imported into the guidance system at the beginning of the experiment. The start and the goal position were identical in both tests, as well as the used OctoMap. In addition, the same RRT-Connect path planner was used with the identical parameters. The planning time was limited to 10 s, the number of planning attempts was limited to 20, and the discretization of robot motion used for collision checking, known as the longest valid segment, was set to 5 cm. This parameters greatly affects the performance of the path planner and the obstacle avoidance. Setting the parameter too low would greatly increase the planning time; however, setting the parameter too high could cause collisions of narrow objects to be missed. Accordingly, 5 cm was chosen knowing that the width of the inserted obstacle is 10 cm. Considering that the execution speed is not of critical importance in our case due to the low velocity of the vehicle, the planning time and the number of planning attempts were increased compared to the experiments in an obstacle-free environment to allow the path planner more time and attempts to plan a safe path. An additional 10 cm padding, or obstacle inflation, was added to the tunnel walls and the obtruding obstacle to add more safety to the vehicle.

Figure 13c shows the side view of the tunnel with an obstacle for easier visualization of the planned and accomplished paths, as well as the distance of the robot to the closest obstacle. Figure 13b depicts in 3D the planned path with the OctoMap representation of the environment. The length of the planned path was 4.12 m in the SIL environment and 4.2 m in HIL. The difference can be explained due to the randomness of the RRT-Connect path planner. The calculated accomplished path in SIL was 4.31 m and 5.17 m in HIL. Similarly to the previous experiment, the bigger difference in the length of the accomplished path and reference path in HIL environment seems to be caused by the noise in the odometry measurement, whereas the SIL experiment used the ground-truth odometry from Gazebo simulator. The RMSD in the HIL test was 0.05 m and 0.007 m in SIL. The duration of the SIL test was approximately 53 s and 67 s for HIL. The vehicle’s mean velocity of 0.1 m/s was equivalent in both experiments; however, higher mean force values were obtained in the SIL test due to the differences in the model of the robot used therein. The difference in the duration of the tests was caused mostly by higher complexity of the system used for HIL test and the latency between different software modules. Table 4 summarizes the key figures for the two tests. As in the previous test, the planning time is not considered critical for our case due to the low velocity of the submersible, and therefore it has not been evaluated.

A number of HIL experiments were included as part of a live demo (The video footage of the demo can be found in https://youtu.be/BYf3hiTCIY8?t=4523) for the UNEXMIN project.

#### 5.2.3. Exploration Experiments

The testing of the exploration software, still in an early stage of development, was performed independently for vertical and horizontal exploration. Vertical exploration was tested in the HIL environment, while horizontal exploration was tested in the SIL environment.

**Figure 14:**
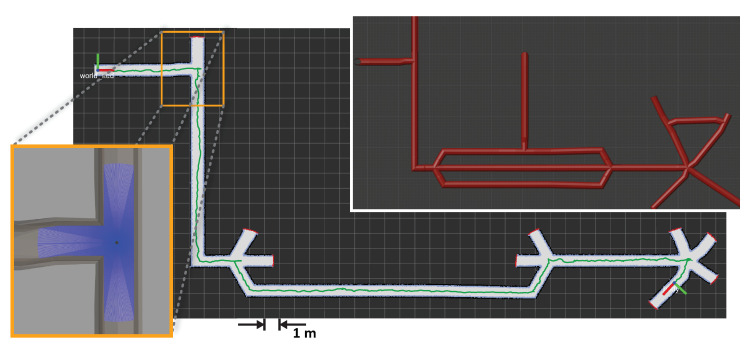
Autonomous horizontal exploration result. Main image: traveled path as green line; detected frontiers as red lines and appearing on the edges of the unexplored tunnels. Overlaid top-right image: model of the mine used for exploration.

In Figure 14, the results of the horizontal exploration, as explained in Section 4.2.2, are depicted. The experiment was performed in the simulation environment, using simplified model of the robot with 360° laser mounted on top of the vehicle. The range of laser range finder was limited to 2 m: its resulting readings are exemplified, for a robot location corresponding to the intersection of mine tunnels, by the bottom-left overlay in Figure 14. The preset requirement of the exploration was chosen as a maximum number of right-most junctions taken. Using a priori knowledge of the map, this parameter was set to 4. No timeout nor maximum distance were set. The model of the mine used for the experiment is depicted in the top-right overlay of Figure 14. Since the focus of this experiment was solely testing the exploration software, we used the robot’s ground-truth position from the Gazebo environment for robot localization to avoid SLAM errors due to poor laser scan matching caused by the mine model’s lack of textures. The experiment was interrupted once the fourth turn was made.

The results represent a successfully completed autonomous horizontal exploration using our merged frontier exploration algorithm with the right-hand rule. The main image in Figure 14 depicts an explored and mapped section of the mine network. The maximum speed of the submersible was limited to 0.2 m/s. The whole experiment took 514 s. The total traversed distance was 77.3 m, while the shortest distance between the start and final location is approximately 56.3 m.

#### 5.2.4. Event Handling Experiments

While exhaustive testing of all failure modes on the final system was difficult to perform due to practical reasons, we successfully tested the handling of a set of both external events, e.g., path replanning due to the detection of new obstacles and internal events, e.g., mechanical component failure and a low-battery level, using our partially-simulated testing paradigms.

##### Path Replanning

**Figure 15:**
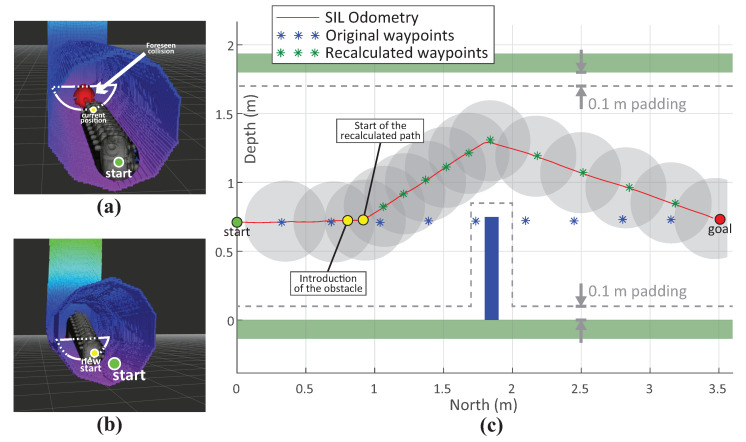
Path replanning experiment depicted in different views. (**a**) Executed path until the moment when the obstacle is introduced and detected. The red color depicts a detected possible collision. (**b**) 3D replanned path from the new start position (position reached in (**a**)). (**c**) Side view of the mine tunnel with the obstacle between start and goal position of the robot. Tunnel walls are depicted in green, the obstacle in blue, and gray circles depicts the final planned path.

In Figure 15, the result of the path replanning due to the detection of a newly introduced obstacle is depicted. The path replanning mechanism is explained in Section 4.2.3. The experiment was performed in the following manner:

- The goal is set at 3.5 m (North) from the start position in a space comprising only the tunnel and no obstacles. The initial path is planned accordingly and is depicted with blue asterisks in the Figure 15c.
- During the executing of the path, the collision checking is recurrently performed by the FCL, checking points of the planned path against the OctoMap of the environment.
- When the vehicle reaches 0.75 m, we introduce a prebuilt OctoMap consisting of a tunnel with an obstacle positioned to block the way to the desired goal destination.
- When FCL detects the possible collision of the initial planned path with the introduced obstacle, the path is declared as invalid and the path execution is aborted. Figure 15a depicts the executed path of the robot until the moment the obstacle is introduced, as well as the possible collision.
- The request for a new path is made.
- The path is recalculated and is depicted with the green asterisks in the Figure 15c. Figure 15b depicts the 3D visualization of the recalculated path as well as the OctoMap of the introduced tunnel and the obstacle.

Figure 15 depicts the side view of the experiment, performed in the SIL environment. The ground-truth odometry readings from Gazebo simulator were used for positioning. The velocity was limited to 0.1 m/s. The obstacle is introduced at a safe distance from the vehicle, namely 1 m, allowing the vehicle to timely and safely pass around it. The time elapsed from the moment the obstacle was introduced to the moment the possible collision was detected was approximately 1.3 s. Then, after 0.7 s the request for the new path was sent, which was planned in approximately 2.3 s. During the first latency of 1.3 s the vehicle kept following the initial path and it traveled 10.2 cm. The whole experimented lasted approximately 205 s. The key figures of the experiment are summarized in Table 5.

##### Low-Battery Handling

**Figure 16:**
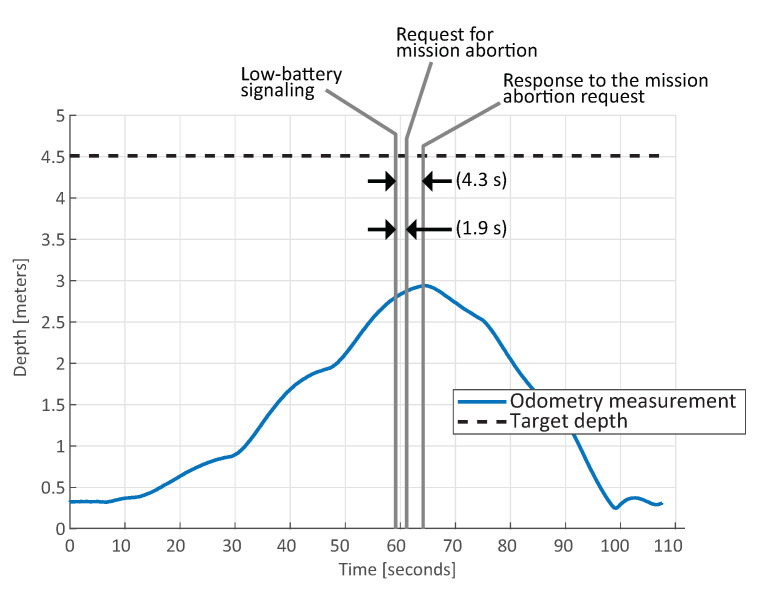
Event handling result.

In Figure 16, the result of the handling of the low-battery signaling in HIL environment is depicted. The goal location of the UX-1 robot was initially sent to the depth of 4.5 m. While descending, the battery level readings were bypassed, triggering the low-battery signal.

This signal was accepted by the Action Executor module, which canceled the mission and sent the new goal location for the UX-1, i.e., the surface. The timing of the triggered signals and the latency of the responses are depicted in the figure. This experiment was included as part of a live demo (The video footage of the demo can be found in https://youtu.be/M1rmX8KjRi0?t=802.) for the UNEXMIN project.

##### Thruster Failure Handling

Furthermore, we validated the handling of a thruster failure in the HIL environment.

We performed three tests with analogous setting but each one with different thruster configuration. The tests were performed in the water tank, without obstacles, performing the maneuver of descending the shaft, entering and traversing the tunnel, and returning to the deployment location. The first part of the experiment, descending the shaft and traversing the tunnel, will be referred to as Part 1, while the second part of the experiment, the return to the deployment location, is denoted as Part 2. The complete reference path was composed of the following 6 waypoints (x,y,z in meters, ψ (yaw) in degrees):

(4) A:(1.0,1.0,2.5,1.0),B:(1.2,1.0,2.5,1.0),C:(3.5,1.0,2.5,1.0),D:(1.5,1.0,2.5,1.0),E:(1.0,1.0,2.5,1.0),F:(1.0,1.0,0.5,1.0),.

**Figure 17:**
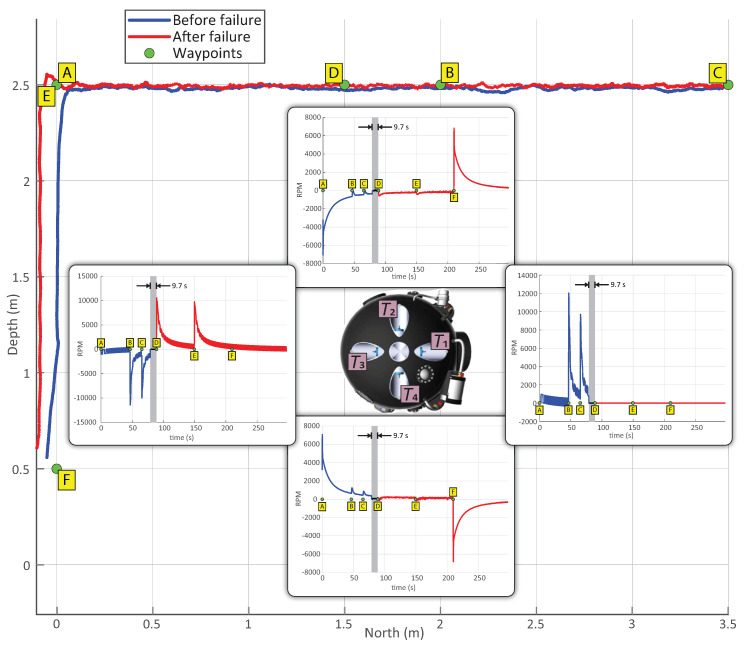
Thruster failure experiment. The main figure depicts the executed trajectory. Yellow squares depicts the moment the corresponding waypoint are sent. The thruster $T_{1}$ failure happens when the waypoint C is reached. The insets represent RPM of four thrusters on the right side of the robot.

The waypoints A, B, and C are considered as the part 1, and D, E, and F the part 2 of the experiment (see Figure 17). The first test, denoted as test I, was performed with all thrusters working. The second test, denoted as test II, was performed with two thrusters disabled. Finally, the third test, denoted as test III, is a hybrid test which was performed with all thrusters working in the part 1, and the part 2 was performed with two thrusters disabled. During the test III, the failure of the thruster $T_{1}$ was simulated by bypassing the output values of the LLC module. The failure was signaled when the waypoint C was reached, which represented the end of part 1. Once the failure was detected and the specific failing thruster identified, the initial thruster allocation matrix, shown in Equation (1), was reconfigured online into

(5) $$B_{new}=\left[\begin{array}{cccccccc} 0 & 0 & -1 & 0 & 0 & 0 & -1 & 0\\ 0 & 1 & 1 & 1 & 0 & -1 & -1 & -1\\ 0 & 1 & 0 & -1 & 0 & 1 & 0 & -1\\ 0 & -l & 0 & l & 0 & l & 0 & -l\\ 0 & 0 & 0 & 0 & 0 & 0 & 0 & 0\\ 0 & 0 & -l & 0 & 0 & 0 & l & 0 \end{array}\right]\;\;.$$

Note that the new matrix has zero values in the columns representing the defective thruster and its symmetric one, that is, $T_{5}$.

For the sake of conciseness, only test III is shown in Figure 17. The odometry measurement is depicted in different colors for the part 1 and part 2 for easier visualization. The RPM of the four thrusters on the right side of the vehicle are shown: due to the symmetry of the vehicle, the equivalent forces are obtained from the thrusters on the other side.

The gray area denoted in the figure represents the latency from the moment of the triggered $T_{1}$ thruster failure to the moment of reinitializing the experiment, once the thruster allocation matrix had been updated. The total latency time between the reception of the thruster failure signal and the resumption of the test was approximately 9.7 s.

The duration of each part of each test is depicted in Table 6a. Table 6b presents the RMSD with respect to the reference path. Attention is drawn to the hybrid test III, whose parts relate to different configurations. It can be noted that, in this test, the path error was very similar for part 1 and part 2; this is expected due to the UX-1 robot being an overactuated system where thrusters are redundant. However, the duration of part 2 in this test was higher: since the RPM limits of the remaining thrusters were not changed after the failure, lower velocities were achieved by the submarine as a consequence of the lower overall thrust achievable.

This experiment confirmed that in the case of a thruster failure the mission can be continued, although at a lower velocity. Higher velocities can be obtained by increasing the RPM limits: however, this is not recommended as it can cause a failure of additional thrusters.

## 6. Conclusions and Future Work

This paper addresses the current state of the autonomous guidance system of the robotic submersible UX-1 developed in the UNEXMIN project, essential software system for the operation of the robot in its target maze-like environments. The corresponding sections detail the solutions adopted in the design and implementation of the guidance system in order to make the system not only effective at its prescheduled tasks but also dynamically responsive to potential asynchronous events, both external, e.g., obstacles, and internal, e.g., low battery or thruster failure. The works presented in this paper stem from the developments disclosed in [7] regarding the guidance and navigation software in an earlier stage of development of the UX-1 robot. However, the structure of the guidance system presented and detailed herein provide a clearer conceptual separation between these subsystems and the tasks they respectively address.

The experimental validation of the implemented guidance system in its integration with the rest of systems of the UX-1 robot is one of the main points of attention of this paper. The experiments presented herein, which involve both fully and partially simulated robot interaction with its environment, prove the capability of the developed guidance system to successfully lead the movement of the submarine in a number of tasks representative of its operation in real mine campaigns. Initial validation experiments following a similar approach were published in [7]: however, this paper extends the experimental settings therein to evaluate in more depth the capabilities of the whole system, as well as the new capabilities of the guidance system presented in this paper, such as the autonomous exploration and event handling. The performed tests, those presented and detailed in this paper and also those, analogous, left outside this paper due to size constrains, address a great variety of situations in terms of structural features of the environment, required tasks, and asynchronous events in which the different modules of the developed guidance system have shown their capabilities.

Despite the satisfactory performance of the implemented guidance system proved by the presented sets of tests and the indubitable usefulness of the used experimental paradigms, a number of questions related to the progressive deployment of the fully autonomous version of the UX-1 submersible in real flooded mines require further clarification. For instance, as indicated in the experimental evaluation section, the scope and validity of the performed hardware-in-the-loop experiments have been hindered by practical aspects such as the limited size of the available physical pool or the need for a connection cable affecting the dynamics of the robot, which deserves additional efforts as future work. Real campaigns in actual abandoned flooded mines appear also of special interest in the path towards full operation of the UX-1, since simulations cannot suffice to illustrate all potential variability of real environments: in this direction, several campaigns in different mines with specific details and peculiarities are already planned, which will lead to an extensive evaluation of the complete platform.

Future works will include the development of a more sophisticated fault tolerance control: precisely in this line of research, an AI-based supervision system based on metacontrol and capable of self-reasoning and self reconfiguration of the submersible resources is currently under development [50]. Additional works will include the integration of horizontal and vertical exploration and its validation in a real mine. In addition, a thorough path planning benchmarking in the HIL environment is planned in order to select the optimal planner for the UX-1 vehicle. The RRT-Connect path planner used in this work showed satisfactory results in terms of length of the planned path and the distance to the obstacle. However, there is room for improvement in the case of path optimization and smoothing and the future works will focus on this. Moreover, path replanning will be further tested and tuned. At the current stage, this has been tested only with the obstacle introduced at sufficient distance and enough time for the UX-1 to safely traverse it. Finally, the performance of the whole guidance system is to be tested with the online mapping capabilities, since at the current stage all the performed experiments used a prebuilt OctoMap imported into the guidance system.

## Figures and Tables

**Table 1:** Characteristics of the UX-1 robot.

Features	Specification
shape	spherical
diameter	0.6 cm
autonomy	5 h
weight in air	106 kg
ballast capacity	2.8 L
max operating depth	500 m
max pressure	50 bar
max speed	0.5 m/s

**Table 2:** Scientific sensors.

Sensor	Parameters
pH	location/time trigger sampling frequency
conductivity	location/time trigger sampling frequency
multispectral unit	location/time trigger distance duration velocity
gamma ray counter	location/time trigger distance duration velocity
sub-bottom profiler	location/time trigger duration
temperature	none (constantly measured)
pressure	none (constantly measured)
magnetic field	location/time trigger sampling frequency
water sampler	location/time trigger
UV fluorescence	location/time trigger exposure time intensity distance

**Table 3:** Key figures of the path planning experiment performed in the water tank. (**a**) Mean and max force reference commands obtained by FL controller. (**b**) Length of the reference path and the accomplished path, RMSD, and the duration of the test.

(**a**)		
**Metrics**	**Value**	**Units**
Mean Fx	1.9	[N]
Mean Fz	1.71	[N]
Max Fx	13.37	[N]
Max Fz	9.42	[N]
(**b**)		
**Metrics**	**Value**	**Units**
Ref. path length	13.4	[m]
Accomp. path length	15.1	[m]
RMSD	0.056	[m]
Duration	203	[s]

**Table 4:** Key figures of the path planning experiment with obstacle avoidance performed in SIL and HIL environments. (**a**) Mean and max force reference commands obtained by the FL controller. (**b**) Length of the reference path and the accomplished path, RMSD, and the duration of the test.

(**a**)			
	**SIL**	**HIL**	
**Metrics**	**Value**		**Units**
Mean Fx	3.1	0.21	[N]
Mean Fz	2.7	0.05	[N]
Max Fx	5.2	0.4	[N]
Max Fz	4.2	0.1	[N]
(**b**)			
	**SIL**	**HIL**	
**Metrics**	**Value**		**Units**
Ref. path length	4.12	4.2	[m]
Accomp. path length	4.31	5.17	[m]
RMSD	0.007	0.05	[m]
duration	53	67	[s]

**Table 5:** Key figures of the path replanning experiment performed in SIL. (**a**) Mean and max force reference commands obtained by FL controller. (**b**) Length of the reference path and the accomplished path, RMSD, and the duration of the test.

(**a**)		
**Metrics**	**Value**	**Units**
Mean Fx	2.13	[N]
Mean Fy	0.12	[N]
Mean Fz	1.14	[N]
Max Fx	3.18	[N]
Max Fy	0.3	[N]
Max Fz	1.93	[N]
(**b**)		
**Metrics**	**Value**	**Units**
Ref. path length	4.24	[m]
Accomp. path length	4.49	[m]
RMSD	0.068	[m]
duration	205	[s]

**Table 6:** The duration of each test per different parts and the RMSD of the vehicle position with respect to the reference trajectory.

(**a**)				
	**Duration**			
**Test**	**Part 1**	**Part 2**	**Complete**	**Units**
I	75	124	199	[s]
II	97	180	277	[s]
III	78	180	258	[s]
(**b**)				
	**RMSD**			
**Test**	**Part 1**	**Part 2**	**Complete**	**Units**
I	0.049	0.051	0.05	[m]
II	0.067	0.073	0.07	[m]
III	0.052	0.059	0.056	[m]

## Abbreviations

The following abbreviations are used in this manuscript:

AUV	Autonomous Underwater Vehicle
DVL	Doppler Velocity Log
GNC	Guidance, Navigation, and Control
HIL	Hardware-In-the-Loop
IMU	Inertial Measurement Unit
LLC	Low-Level Control
RMSD	Root-Mean-Square Deviation
ROS	Robot Operating System
ROV	Remotely Operated Vehicle
SF	Sensor Fusion
SIL	Software-In-the-Loop
SLAM	Simultaneous Localization and Mapping
SLS	Structured Light System
UNEXMIN	UNderwater EXplorer for flooded MINes
